# Multi‐Omics Integration Reveals That SN‐011 Targets JUNB to Upregulate ADGRE5 and Restore Vascular Endothelial Cell Communication in Ischemic Stroke

**DOI:** 10.1111/cbdd.70363

**Published:** 2026-08-04

**Authors:** XiangLing Ou, ZunKe Gong

**Affiliations:** ^1^ The Affiliated Xuzhou Rehabilitation Hospital of Xuzhou Medical University Xuzhou Jiangsu Province China

**Keywords:** ADGRE5, ischemic stroke, JUNB, SN‐011

## Abstract

Ischemic stroke (IS) is a cerebrovascular disease with high mortality and disability rates, currently lacking effective therapeutic targets. The STING inhibitor SN‐011 shows potential in IS treatment, but its mechanism of action remains unclear. This study aims to explore the key molecular mechanisms of SN‐011 in treating IS through bioinformatics approaches. IS transcriptome datasets were analyzed to identify differentially expressed genes. Mendelian randomization using brain eQTL and IS‐GWAS data identified genes with causal relationships to IS. Single‐cell transcriptome, pseudo‐time trajectory, intercellular communication, and transcription factor regulatory network analyzes were performed. Molecular docking and DARTS‐WB assay validated SN‐011 binding to transcription factors. Transcriptomic analysis identified 77 intersecting genes. Mendelian randomization revealed ADGRE5 as a protective gene for IS (OR < 1), significantly downregulated in venous endothelial cells (vECs) during disease progression. Cell communication analysis showed ADGRE5‐high vECs interact with immune, glial, and stromal cells via LAMININ (Lamb2‐CD44, Lamb2‐Itga6+Itgb1, Lamb2‐Dag1 pair) and JAM signaling pathways. Transcription factor analysis identified JUNB as a negative regulator of ADGRE5. Molecular docking (−6.8 kcal/mol) combined with an in vitro DARTS‐WB assay confirmed the interaction between SN‐011 and JUNB. In OGD‐induced endothelial cell injury models, SN‐011 suppressed JUNB expression, restored ADGRE5 expression inhibited by JUNB overexpression, reversed the downregulation of LAMB2 and CD44, and reduced the expression of the pro‐inflammatory cytokines IL‐6 and IL‐1β. Notably, blockade of LAMB2 largely abolished these protective effects, indicating that the anti‐inflammatory and endothelial‐protective activities of SN‐011 are mediated, at least in part, through restoration of the LAMB2‐CD44 signaling axis. ADGRE5 downregulation in vECs may impair vascular repair by disrupting LAMININ‐mediated intercellular communication. SN‐011 may exert neuroprotective effects by targeting JUNB to upregulate ADGRE5 expression and restore the vEC‐centered cellular communication network, providing a theoretical basis for SN‐011 as a potential IS therapeutic.

## Graphical abstract

Ischemic stroke (IS) remains a leading cause of disability with limited therapeutic targets. Using multi‐omics integration, we identified ADGRE5 as a protective gene significantly downregulated in venous endothelial cells (vECs) during IS. JUNB acts as a key upstream transcription factor that negatively regulates ADGRE5 expression. The STING inhibitor SN‐011 directly targets JUNB, thereby upregulating ADGRE5 and restoring vEC‐centered intercellular communication via the LAMININ/JAM signaling axis. This regulatory cascade repairs vascular function, suppresses neuroinflammation, and exerts neuroprotection in ischemic stroke, highlighting SN‐011 as a promising therapeutic candidate.

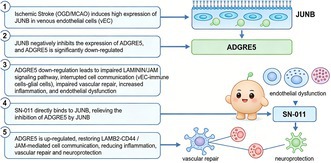

## 1 Introduction

Ischemic stroke is one of the most severe neurovascular diseases (Chugh 2019; Hurford et al. 2020). It accounts for 80%–87% of all stroke cases, primarily caused by thrombus or embolus obstructing cerebral blood vessels, leading to restricted cerebral blood flow (Benjamin et al. 2018; Rosamond et al. 2008). Ischemia in any part of the brain results in long‐term disability (loss of neurological function) and high mortality (Feigin et al. 2014; Lozano et al. 2012; Warlow et al. 2003).

The pathophysiology of ischemic stroke is influenced by a complex interplay of factors, among which genetic predisposition plays a critical role. Recent studies have identified several key genetic markers associated with both the occurrence and prognosis of IS, including LAMC1, DIAPH1, and ATP2B1 (Surakka et al. 2023; Ren et al. 2020; Mishra et al. 2022; Söderholm et al. 2019). These findings highlight that molecular mechanisms underlying genetic susceptibility are fundamental to disease onset and recovery. However, current therapeutic strategies for ischemic stroke primarily rely on mechanical or pharmacological (thrombolytic) approaches to reopen blocked blood vessels—interventions that do not target these molecular pathways. Existing thrombolytic therapies such as RT‐PA have significant limitations, including a narrow therapeutic window (being most effective when administered within 6 h of stroke onset), the risk of intracerebral hemorrhage, and the fact that only a minority of patients benefit from them (Ning et al. 2010). Therefore, deepening our understanding of the molecular pathophysiology of ischemic stroke and identifying novel intervention targets are crucial for developing more effective therapeutic strategies.

Understanding the complex pathophysiological mechanisms underlying ischemic stroke is crucial for exploring and developing novel interventions. Ischemic stroke triggers a multifaceted cascade of events, including cellular energy depletion, excitotoxicity, oxidative stress, and neuroinflammation (Jiang et al. 2021). Neuroinflammation, in particular, is recognized as one of the key contributors to ischemic brain injury. This multifaceted process primarily manifests as the activation of immune cells, predominantly microglia, within the brain environment. While microglial activation is essential for tissue repair and remodeling, excessive activation exacerbates neuronal cell death. The cyclic GMP‐AMP synthase (cGAS)‐stimulus‐triggered interferon genes (STING) signaling pathway has been identified as a key mediator of microglial activation and neuroinflammation following ischemic stroke. This pathway is activated by mitochondrial DNA accumulation, ultimately triggering the transcription of interferon (IFN)‐stimulated genes and several proinflammatory cytokines (Dai et al. 2019; Li et al. 2018, 2023; Liu et al. 2022; Paul et al. 2021; Mathur et al. 2017). Subsequently, downstream pathway networks regulating gene expression are activated, involving lipid metabolism, inflammation, autophagy, apoptosis, and cellular senescence (Akhmetova et al. 2021; Liu et al. 2019; Zhao et al. 2018; Gulen et al. 2017; Glück and Ablasser 2019; Lan et al. 2019; Abdisalaam et al. 2020). Studies in ischemia–reperfusion models have revealed that the STING‐GPX4 axis promotes ferroptosis in cardiomyocytes and forms a positive feedback loop. Knockout of STING suppresses oxidative stress, reduces ferroptosis, and mitigates I/R injury (Wang et al. 2025).

SN‐011 is an effective mouse and human STING inhibitor identified through computer‐aided docking screening of over 500,000 lead‐like compounds against the crystal structure of the human STING C‐terminal domain (Hong et al. 2021). SN‐011 functions as a direct competitive antagonist of STING (Chang et al. 2023). It binds to the CDN‐binding pocket of the STING dimer with higher affinity than the endogenous agonist 2′3′ cGAMP (Hong et al. 2021). By occupying this pocket, SN‐011 locks STING in an open, inactive conformation (Hong et al. 2021). This prevents the conformational change required for STING activation, thereby inhibiting all downstream signaling events. Relevant studies indicate SN‐011 can suppress inflammatory cytokine expression, reduce inflammatory responses, improve survival rates, and alleviate renal dysfunction (Li et al. 2025). However, the mechanism of action of SN‐011 in ischemic stroke remains unclear. Specifically, whether SN‐011 exerts neuroprotective effects by regulating molecular networks in specific cell types remains to be elucidated. Furthermore, although the STING pathway in ischemic stroke has garnered extensive attention, existing studies predominantly focus on immune cells. Its function within vascular endothelial cells and its regulation of intercellular communication networks remain poorly understood.

Based on this, this study integrated transcriptomic differential analysis, Mendelian randomization, single‐cell transcriptomic sequencing, pseudo‐time analysis, cellular communication network construction, transcription factor regulatory network analysis, and molecular docking technology to systematically investigate the potential molecular mechanisms underlying SN‐011 treatment for ischemic stroke. We focused on deciphering the expression characteristics and regulatory network of ADGRE5 in cerebral vascular endothelial cells, revealing that SN‐011 may target JUNB to upregulate ADGRE5 expression. This, in turn, reconstructs the vEC‐immune cell‐glial cell communication network centered on the LAMININ and JAM pathways, ultimately promoting vascular repair and neuroprotection. This study provides novel theoretical support for SN‐011's therapeutic efficacy in ischemic stroke and pioneers new therapeutic approaches targeting vascular endothelial cells.

## 2 Materials and Methods

### 2.1 Data Acquisition

1. Transcriptome data: Downloaded the ischemic stroke (IS) transcriptome dataset GSE236006 from the GEO database (https://www.ncbi.nlm.nih.gov/geo/browse/). The data originates from mice and is classified as high‐throughput sequencing data. Select nine samples from this dataset: three sham‐operated mice, three transient middle cerebral artery occlusion (tMCAO) mouse models, and striatal sequencing data from mice treated with the STING inhibitor SN‐011 for subsequent differential analysis. The independent GEO dataset GSE183439 (containing 4 sham‐operated samples and 4 stroke model samples) was used for external validation.
2. The data for MR Analysis: The eQTL data obtained from the GTEx database (https://gtexportal.org/home/). We utilized eQTL data from three brain tissues associated with the striatum in the GTEx v10 dataset: the caudate nucleus (Caudate basal ganglia), nucleus accumbens (Nucleus accumbens basal ganglia), and putamen (Putamen basal ganglia). The GWAS data for ischemic stroke were sourced from the IEU database (https://opengwas.io/), comprising 3314 European‐ancestry cases and 357,880 European‐ancestry controls, with ID ukb‐d‐I9_STR_EXH.
3. Single‐Cell Data: Single‐cell data for ischemic stroke were obtained from the GSE174574 dataset in the GEO database. This dataset comprises six samples: three from middle cerebral artery occlusion (MCAO) mouse models and three from sham‐operated mice.

### 2.2 Transcriptome Analysis

Annotation files for mice were downloaded from the Ensembl database (https://asia.ensembl.org/index.html?), and protein‐coding genes were filtered. Ensemble IDs in the dataset were converted to Symbol IDs. The edgeR package (ver 4.6.3) in R software (ver 4.5.0) was used to calculate differentially expressed genes (DEGs) between the ischemic stroke group and healthy control group (tMCAO vs. sham) and between the ischemic stroke group and SN‐011‐treated group (tMCAO vs. SN‐011) in the transcriptome dataset GSE236006. Using the screening criteria of *p*‐value < 0.05 and |log2FC| > 0.58. The threshold for differential analysis of the externally validated GEO dataset GSE183439 is the same as above (*t*‐test).

### 2.3 MR Analysis

The significance threshold for instrumental variables was set at *p*_val < 1e‐05. Considering that linkage disequilibrium (LD) between SNPs could introduce bias, we required LD with exposure‐associated SNPs to satisfy *r* ^2^ < 0.001 and kb = 10,000. To assess sample overlap effects and weak instrumental bias, we calculated F‐statistics and retained only SNPs with *F* > 10. This approach helps mitigate bias from weak instrumental variables, enhancing the reliability of our findings. Additionally, to exclude rare variants from influencing results, we excluded SNPs with MAF < 0.01.

The TwoSampleMR package (ver 0.6.22) was used for TSMR analysis. When nSNP ≥ 2, the inverse variance weighted (IVW) method was primarily employed to determine causality between exposure and outcome. When nSNP = 1, the Wald ratio method was primarily used to establish causality. Sensitivity analyzes employed two methods: heterogeneity testing and horizontal pleiotropy testing. Heterogeneity was assessed using Cochrane's *Q* test, with Q_*p*_val > 0.05 indicating no heterogeneity. Horizontal pleiotropy was evaluated via the MR‐Egger intercept test, where *p*_val > 0.05 signified no horizontal pleiotropy.

### 2.4 Single‐Cell Data Acquisition and Processing

This dataset employed the following quality control criteria to remove low‐quality cells: gene count per cell > 200 and < 3000, mitochondrial‐related gene expression proportion < 10%, and filtering out genes expressed in fewer than three cells. Data from the six samples were integrated using the Seurat package (ver 5.3.0) in R software (ver 4.5.0) and organized into a Seurat object for subsequent single‐cell data processing. Raw data were normalized using the NormalizeData function, followed by identification of highly variable genes, with the threshold set at 2000. Next, the decontX function was used to calculate and predict the extent of environmental free RNA contamination per cell, removing low‐quality cells with contamination rates > 0.2. The RunHarmony function was then applied to perform batch normalization across all samples. The ScaleData function scaled the data and corrected for the influence of mitochondrial‐related genes.

The top 2000 most variable genes across all samples were input into the RunPCA function to compute principal component analysis (PCA) dimensions. The first 20 PCA dimensions were selected, and the FindClusters function—a clustering algorithm optimized using a shared nearest neighbor (SNN) graph—was applied for clustering analysis. The clustree package (ver 0.5.1) was used to generate a clustering tree for visualizing and evaluating cell clustering results at multiple resolutions, with a resolution of 0.7 selected. Finally, the clustering results were visualized using the RunUMAP function with default parameters and the top 20 PCA dimensions.

Marker genes for various cell types in the brain were sourced from the CellMarker platform (http://bio‐bigdata.hrbmu.edu.cn/CellMarker/) and results from previous studies (PMID: 41575950 and 34496660). Differential cell‐type composition analysis was performed using the propeller method. To account for the compositional characteristics of cell proportion data, a centered log‐ratio (CLR) transformation was applied to alleviate the constant‐sum constraint. Statistical testing was conducted within the limma framework using empirical Bayes moderation, which improves the stability of variance estimation and enhances statistical power, particularly in datasets with a limited number of biological replicates. The FindMarkers function was applied with parameters logfc.threshold = 0.25 and min.pct = 0.1 to perform differential analysis between the MCAO and sham groups for each cell type, identifying key genes exhibiting significant differences in specific cell types.

### 2.5 Functional Enrichment Analysis

To investigate the biological functions and potential mechanisms of differentially expressed genes in capEC and vEC, we performed Gene Ontology (GO) enrichment analysis. GO annotation data for the relevant species was obtained from the Gene Ontology Consortium (http://geneontology.org/). The analysis was conducted using the clusterProfiler package (version 4.16.0) in R/Bioconductor, which implements hypergeometric distribution‐based statistical tests to identify significantly enriched GO terms. Enrichment analysis was performed across three GO categories: biological process (BP), cellular component (CC), and molecular function (MF). To account for multiple testing, *p*‐values were adjusted using the Benjamini‐Hochberg (BH) method to control the false discovery rate (FDR). GO terms with adjusted *p*‐value < 0.05 were considered statistically significantly enriched.

### 2.6 Pseudo‐Sequential Analysis

Perform pseudo‐time analysis of key cell types using the monocle2 package (ver 2.36.0) in R software (ver 4.5.0). The input data required to create a cds object via the newCellDataSet function are: (1) the gene‐cell matrix of raw count data from Seurat‐processed single‐cell data; (2) the meta.data from the single‐cell data, created as an S4 object using the new function; (3) a data frame storing gene names, created as an S4 object using the new function. The expression data within the cds object is then normalized, and genes expressed in fewer than 10 cells are filtered out. For trajectory analysis, cells are pseudo‐time‐ordered using genes meeting the Monocle2 identification thresholds: mean_expression ≥ 0.01 and dispersion_empirical ≥ 1 * dispersion_fit. Dimension reduction is then performed using the reduceDimension function with parameters reduction_method = “DDRTree” and max_components = 2 (Mao et al. 2015). The plot_cell_trajectory function is subsequently applied to sort and visualize the cells. Furthermore, to determine the initial direction of the proposed temporal trajectory, we anchored the root cell to a normal cell based on biological prior knowledge. We calculated the centroid of the normal cells in the reduced‐dimensional space and designated the trajectory node closest to this centroid as the root cell (pseudo‐time = 0). This approach reflects the biological process central to this study: the progressive differentiation of cells from a normal state to a disease state, along with alterations in key cellular functions.

### 2.7 Cell Communication

To quantify the communication networks between key cell types and other cells, we employed the CellChat package (ver 2.2.0) to investigate their underlying cellular communication. First, single‐cell data from disease groups were extracted. Key cell types were divided into high‐ and low‐expression groups based on median expression levels of key genes. In exploratory single‐cell analysis, when no a priori biological thresholds are available, the median is a commonly used method for binarizing continuous variables. Its rationale lies in the following: (1) Robustness: The median is insensitive to outliers and is well‐suited to the skewed distributions commonly observed in single‐cell expression data; (2) Balance between groups: Median‐based partitioning ensures that the high‐ and low‐expression groups have equal sample sizes, thereby avoiding a decline in statistical power due to a significant disparity in sample sizes; (3) Reproducibility: As a measure of central tendency, the median exhibits good stability across different datasets. Similar median‐based grouping strategies have been widely adopted in single‐cell research. Subsequently, the gene‐cell matrix and cell annotation information from the standardized data were extracted. The “createCellChat” function was used to create a CellChat object, and the mouse ligand‐receptor database provided by CellChat was imported. High‐expression genes were identified, and ligand‐receptor pairs for these genes were screened and identified. The computeCommunProb function estimated communication probabilities for cell interactions based on gene expression values. Subsequently, the filterCommunication function filtered out communication relationships between low‐quality cells using the parameter min.cell = 10. Finally, CellChat objects from each group were merged, and the netVisual_bubble function visualized the results.

### 2.8 Transcription Factor Analysis

To infer transcription factors regulating key genes, we employed Python (ver 3.8) and the pySCENIC package (ver 0.12.1) to assess transcription factor enrichment and regulator activity. First, we inferred gene–gene co‐expression networks between transcription factors (TFs) and their potential targets using the GRNboost2 (Aibar et al. 2017) algorithm. Next, we performed regulator prediction steps, with motif annotation database files sourced from https://resources.aertslab.org/cistarget. Finally, we analyzed single‐cell transcriptomic data using the AUCell algorithm to evaluate the activity scores of each regulator. Next, we visualized the analysis results using the plotRSS function in the SCENIC package (ver 1.3.1) within R software, with parameters zThreshold = 0.3 and thr = 1. To validate the reliability of transcription factor regulatory relationships, we conducted cross‐validation using the ChEA database. ChEA integrates ChIP‐seq data from projects such as ENCODE and Roadmap Epigenomics, providing experimentally supported TF‐target gene regulatory relationships. Using ADGRE5 as the target gene, we queried the comprehensive scores of all transcription factors in ChEA; lower scores indicate stronger evidence.

### 2.9 Molecular Docking

Retrieve key genes from the UniProt database (https://www.uniprot.org/), select the human species, obtain the corresponding UniProt IDs, and then download the protein structure files predicted by the AlphaFold database in the Structure section. Subsequently, download the 3D structure file of the STING inhibitor SN‐011 from the PubChem database (https://pubchem.ncbi.nlm.nih.gov/).

First, proteins were dehydrated and small molecules removed using PyMOL (ver 3.0.4). Hydrogen atoms were added to proteins using Autodock (ver 1.5.7) to ensure protonation of all amino acids. After designating the protein as the receptor, the active site position was identified using the online tool Prankweb (https://prankweb.cz/). A grid box was specified within Autodock to define the docking region. Hydrogen atoms were then added to the drug molecule, which was set as the ligand. Rotation bonds were checked and selected. Finally, generate the config file, perform 20 docking runs using Autodock vina (ver 1.1.2), and visualize the results with Pymol.

### 2.10 Experimental Validation

#### 2.10.1 Cell Culture and Oxygen–Glucose Deprivation Model Establishment

Primary human brain microvascular endothelial cells (HBMECs) were purchased from Shanghai Jinyuan Biotechnology Co. Ltd. (Product No.: JY‐J140; Product Name: Human Primary Brain Microvascular Endothelial Cells; Product Link: https://ssrcc.com.cn/product/?id=885) and routinely cultured in endothelial cell medium (ECM) containing 10% fetal bovine serum (FBS), 1% endothelial cell growth supplement, and 1% penicillin/streptomycin at 37°C with 5% CO_2_. All experiments were performed using cells at passages 3–6.

The oxygen–glucose deprivation (OGD) model was used to simulate ischemic injury in vitro. The specific procedure was as follows: cells were cultured in glucose‐free DMEM medium and placed in a hypoxic incubator (95% N_2_, 5% CO_2_) for 4 h, after which the medium was replaced with normal medium, and cells were further cultured under normoxic conditions for 24 h prior to subsequent assays. Control group cells were cultured in normal medium without OGD treatment.

#### 2.10.2 Drug Treatment and Genetic Intervention

The STING inhibitor SN‐011 (Source: Cat. No.: HY‐145010; Product Name: SN‐011; MedChemExpress (MCE); https://www.medchemexpress.cn/sn‐011.html) was dissolved in dimethyl sulfoxide (DMSO) to prepare a stock solution. For experiments, it was diluted with culture medium to final concentrations of 0.1, 1, and 10 μM and added to the cell culture system for 24 h after OGD treatment.

The JUNB overexpression plasmid (JUNB‐OE) and negative control plasmid (NC) were constructed by Shanghai Genechem Co. Ltd. Transfection was performed according to the Lipofectamine 3000 (Invitrogen, USA) manufacturer's instructions, and cells were collected 48 h after transfection for subsequent experiments. For the JUNB‐OE combined with SN‐011 treatment group, SN‐011 (10 μM) was added 24 h after transfection and further incubated for 24 h.

The LAMB2 blocker (anti‐LAMB2 neutralizing antibody) was purchased from Sigma‐Aldrich (Cat. No.: SAB4501727) and added to the co‐culture system at a final concentration of 5 μg/mL in the OGD+SN‐011 treatment group for 24 h.

#### 2.10.3 Neutrophil and Vascular Endothelial Cell co‐Culture System

Neutrophils were obtained from mouse neutrophils (Product No.: SNP‐M207; Product Link: https://www.sunncell.com.cn/product/SNP‐M207). The co‐culture system of HBMECs and neutrophils was established using a Transwell system (0.4 μm pore size, Corning, USA). HBMECs were seeded in the lower chamber, and neutrophils were seeded in the upper chamber at 5 × 10^5^ cells/well. After co‐culture for 24 h, cells were collected for analysis.

#### 2.10.4 Total RNA Extraction and Real‐Time Quantitative PCR (qPCR)

Total RNA was extracted from cells using TRIzol reagent (Invitrogen, USA). RNA concentration and purity were determined using a NanoDrop 2000 spectrophotometer. A total of 1 μg of RNA was reverse‐transcribed into cDNA using PrimeScript RT Master Mix (Takara, Japan). qPCR was performed using TB Green Premix Ex Taq II (Takara, Japan) on a LightCycler 480 II Real‐Time PCR System (Roche, Switzerland). The reaction conditions were as follows: pre‐denaturation at 95°C for 30 s, followed by 40 cycles of denaturation at 95°C for 5 s and annealing/extension at 60°C for 30 s. The relative expression levels of target genes were calculated using the 2^−ΔΔCt^ method, with GAPDH as the internal reference. The primer sequences used are listed in Table 1.

**TABLE 1:** qPCR primer sequences.

Gene	Forward primer (5′ → 3′)	Reverse primer (5′ → 3′)
JUNB	5′‐cagctgccggctggctgcta‐3′	5′‐caacggcgtgatcacgacga‐3′
ADGRE5	5′‐acacagaggggagctacgac‐3′	5′‐taaaagctacggcacctgcg‐3′
LAMB2	5′‐gctggctgccacactggcac‐3′	5′‐tcgcatccagaatgtagtca‐3′
CD44	5′‐ctccacctgaagaagattgt‐3′	5′‐acatcagtcacagacctgcc‐3′
IL‐6	5′‐gagaagattccaaagatgta‐3′	5′‐gagacatgtaacaagagtaa‐3′
IL‐1β	5′‐tgttcctgaactcaactgtg‐3′	5′‐tgcaaagtctggcttggaag‐3′
GAPDH	5′‐acccatggcaaattccatgg‐3′	5′‐gtcatgggtgtgaaccatga‐3′

#### 2.10.5 Western Blot Assay

Cells were lysed in RIPA buffer supplemented with protease and phosphatase inhibitors. Protein concentrations were determined by BCA assay. Equal amounts of protein were separated by SDS‐PAGE and transferred onto PVDF membranes. Membranes were blocked and then incubated overnight at 4°C with the following primary antibodies: anti‐JUNB (Thermo Fisher, #701702), anti‐ADGRE5/CD97 (Proteintech, #84255‐6‐RR), anti‐β‐actin (Proteintech, #66009‐1‐Ig), anti‐LAMB2 (Proteintech, #30943‐1‐AP), anti‐CD44 (Proteintech, #15675‐1‐AP), anti‐IL‐6 (Proteintech, #21865‐1‐AP), and anti‐IL‐1β (Thermo Fisher, #710331). After washing, membranes were incubated with HRP‐conjugated secondary antibodies. Protein bands were visualized by enhanced chemiluminescence and quantified by densitometry. β‐actin served as the loading control.

For assessment of JUNB‐ADGRE5 regulation, HBMECs were transfected with a JUNB overexpression plasmid using Lipofectamine 3000 for 48 h. In the combined treatment group, 10 μM SN‐011 was added 24 h post‐transfection and maintained for an additional 24 h. For co‐culture experiments, HBMECs were seeded in the lower chamber and neutrophils in the upper chamber of a Transwell system. After OGD/R treatment, cells were incubated with DMSO, 10 μM SN‐011, or SN‐011 plus anti‐LAMB2 neutralizing antibody (5 μg/mL) for 24 h. HBMECs were then collected for LAMB2 analysis, while neutrophils were collected for CD44, IL‐6, and IL‐1β analysis.

#### 2.10.6 DARTS‐WB Assay

HBMECs subjected to OGD/R were lysed in non‐denaturing buffer (50 mM Tris–HCl pH 7.5, 150 mM NaCl, 5% glycerol, 0.5% NP‐40 or Triton X‐100) without protease inhibitors. After centrifugation, protein concentration was quantified by BCA assay. Lysates were pre‐incubated with DMSO, 10 μM SN‐011, or 50 μM SN‐011 for 30 min at room temperature (final DMSO ≤ 0.5%). The optimal Pronase concentration was predetermined by pilot titration to yield ~30%–50% residual JUNB in the DMSO control group. Samples were then digested with Pronase at the optimized ratio for 10–15 min at room temperature. Reactions were terminated by adding 4× SDS loading buffer and boiling at 95°C for 10 min. Proteins were resolved by SDS‐PAGE, transferred to membranes, and probed with anti‐JUNB and anti‐β‐actin antibodies. Band intensities were quantified and normalized to β‐actin. Protection of JUNB by SN‐011 was expressed as the relative intensity compared to the undigested DMSO control.

#### 2.10.7 Statistical Analysis

All experiments were independently repeated at least three times, and the results are presented as mean ± standard deviation (mean ± SD). Statistical analysis was performed using GraphPad Prism 9.0 software. Comparisons among multiple groups were conducted using one‐way analysis of variance (one‐way ANOVA), followed by Tukey's post hoc test. Comparisons between two groups were performed using Student's *t*‐test. *p* value < 0.05 was considered statistically significant.

## 3 Results

### 3.1 Screening of IS‐Associated Genes and Causal Inference

**FIGURE 1:**
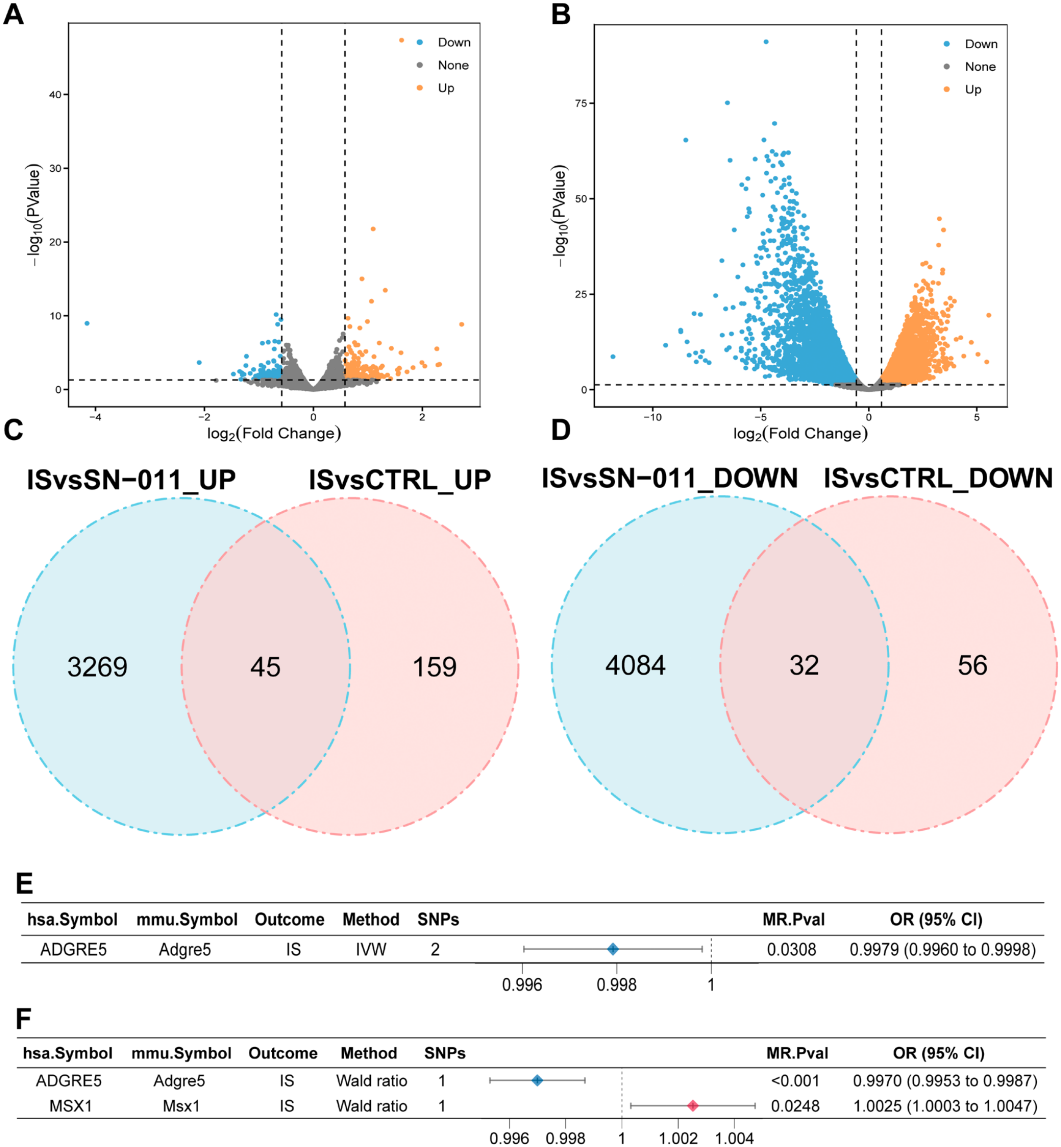
Screening and Causal Inference of Candidate Genes for Ischemic Stroke. (A) Differential analysis results between tMCAO and sham; (B) Differential analysis results between tMCAO and SN‐011; (C) Intersection of differentially up‐regulated genes; (D) Intersection of differentially down‐regulated genes; (E) eQTL data from the caudate nucleus and MR analysis results for ischemic stroke; (F) eQTL data from the nucleus accumbens and MR analysis results for ischemic stroke.

To identify novel intervention targets for ischemic stroke, our study first conducted principal component analysis (PCA) (Figure S1) and differential analysis based on IS sequencing data. Differential analysis results (Figure 1A,B) revealed 292 differentially expressed genes between tMCAO and sham, comprising 204 upregulated genes and 88 downregulated genes. The comparison between tMCAO and SN‐011 identified 7430 differentially expressed genes, including 3314 upregulated genes and 4116 downregulated genes. Subsequently, the intersections of the up‐ and down‐regulated differentially expressed genes from both analyzes were identified, ultimately yielding 77 intersecting genes (45 IS_up genes, 32 IS_down genes) (Figure 1C,D).

To further identify genes with causal relationships to IS, MR analysis was performed using caudate nucleus eQTL data corresponding to the 77 differentially expressed genes. Using a significance threshold of *p* < 0.05, combined with the screening criteria of no significant heterogeneity (*Q*‐value > 0.05) and no pleiotropy bias (*p* > 0.05), one gene (ADGRE5) was identified as having a potential causal relationship with IS (Figure 1E). This gene showed a negative association with IS (OR < 1), indicating that decreased ADGRE5 expression increases IS risk, which is consistent with the direction of the differential expression analysis results. Using eQTL data from the nucleus accumbens for MR analysis, two genes (ADGRE5 and MSX1) were identified as potentially causally related to IS (Figure 1F). Among these, ADGRE5 showed a negative correlation with IS (OR < 1), meaning reduced gene expression increases IS risk; MSX1 showed a positive correlation with IS (OR > 1), indicating that increased gene expression elevates IS risk, both consistent with the direction of the differential findings. MR analysis using eQTL data from the nucleus accumbens did not identify genes with potential causal relationships to IS.

Ultimately, based on comprehensive evidence from datasets of the caudate nucleus, nucleus accumbens, and shell nucleus, ADGRE5 and MSX1 were identified as two key genes for subsequent studies.

### 3.2 Single‐Cell Analysis Identifies Adgre5 in capEC and vEC and Its Functional Enrichment

**FIGURE 2:**
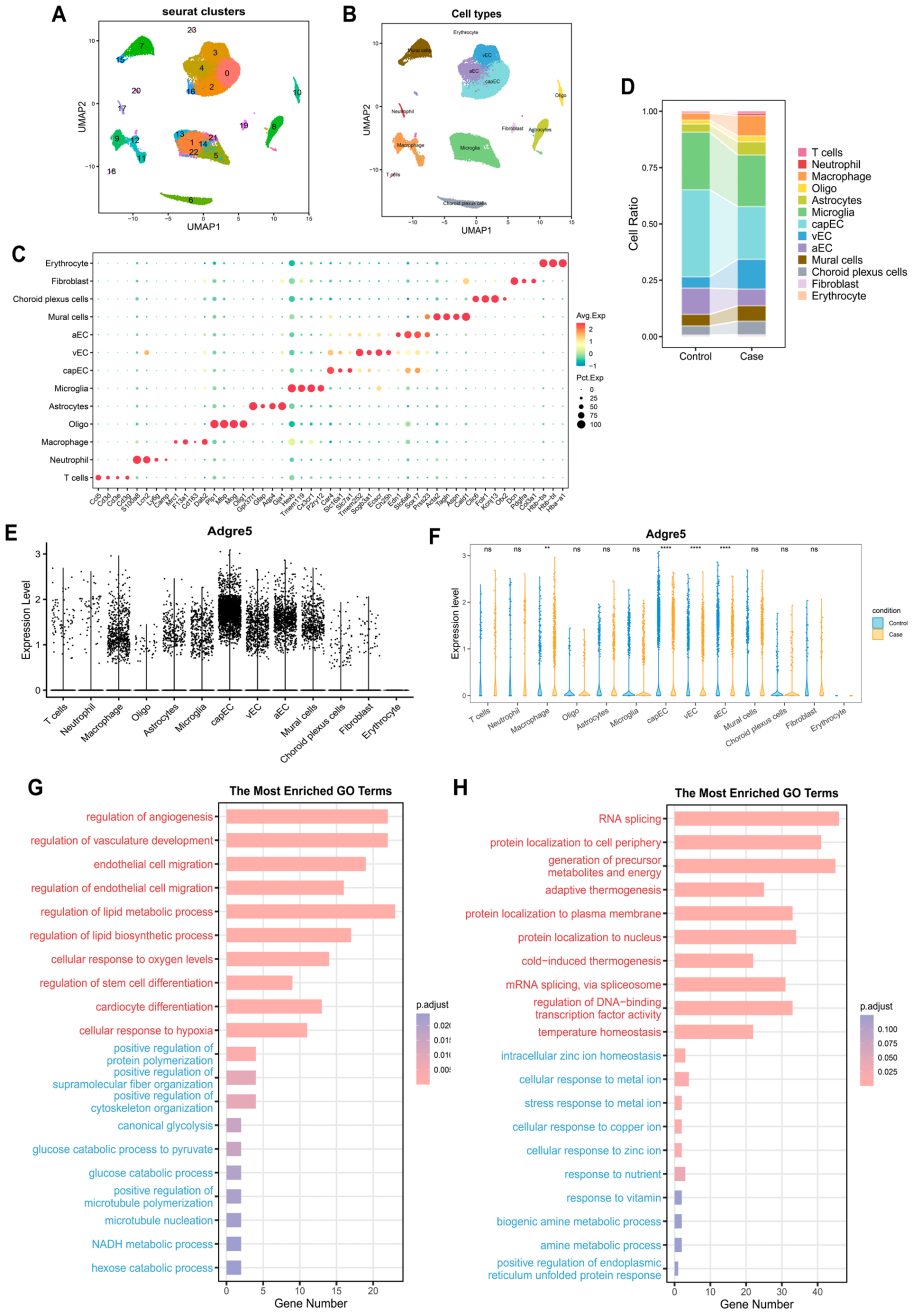
Single‐Cell Transcriptomic Landscape of IS and Functional Enrichment Analysis of Adgre5 in capEC and vEC. (A) UMAP plot of cell clustering results at resolution 0.7; (B) UMAP plot of 13 cell types; (C) Bubble plot of marker gene expression for each cell type; (D) Distribution map of different cell proportions between ischemic stroke patients and the control group; (E) Overall expression of Adger5 in different cell types; (F) Differences in Adger5 expression between ischemic stroke models and control groups across various cell types; (G) Functional enrichment results for Adgre5 in capEC; (H) Functional enrichment results for Adgre5 in vEC (Red indicates enrichment of differentially up‐regulated genes; blue indicates enrichment of differentially down‐regulated genes).

After filtering out low‐quality cells with gene counts < 200 and > 3000, as well as those with mitochondrial‐related gene expression < 10%, we ultimately obtained 51,387 deeply sequenced cells. After PCA dimensionality reduction, the samples exhibited clear separation by batch, indicating a strong batch effect. Following Harmony‐based batch removal, samples from different batches were uniformly mixed, demonstrating that the batch effect had been effectively eliminated (Figure S2). Selecting a resolution of 0.7 yielded a total of 24 cell clusters (Figure 2A). By defining individual clusters through comparison with known lineages or classical marker genes, these 24 clusters were annotated into 13 cell types (Figure 2B,C): T cells (Ccl5, Cd3d, Cd3e, Cd3g), neutrophils (S100a8, Lcn2, Ly6g, Camp), macrophages (Mrc1, F13a1, Cd163, Dab2), oligodendrocytes (Plp1, Mbp, Mog, Olig1), astrocytes (Gpr37l1, Gfap, Aqp4, Gja1), microglia (Hexb, Tmem119, Cx3cr1, P2ry12), capillary endothelial cells (Car4, Slc16a1, Slc7a1), venous endothelial cells (Tmem252, Scgb3a1, Ecscr, Ch25h), arterial endothelial cells (Edn1, Slc6a6, Sox17, Prss23), parietal cells (Acta2, Tagln, Aspn, Cald1), choroid plexus cells (Clic6, Folr1, Kcnj13, Otx2), fibroblasts (Dcn, Pdgfra, Col3a1), and erythrocytes (Hbb‐bs, Hbb‐bt, Hba‐a1). The cell distribution map revealed that macrophages, astrocytes, vascular endothelial cells (vECs), parietal cells, and choroidal plexus cells were significantly enriched in the disease group, whereas capillary endothelial cells (capECs) and arterial endothelial cells (aECs) exhibited higher proportions in the normal group (Figure 2D). The propeller test results further corroborated these descriptive findings (Table S1).

To further identify key candidate targets, we compared the expression patterns of Msx1 and Adgre5 across all cell types between the MCAO and sham groups (Table 2). The results showed that Msx1 was significantly differentially expressed only in choroid plexus cells; however, its expression trend (logFC < 0) was inconsistent with the findings from bulk transcriptomic and Mendelian randomization (MR) analyzes. In contrast, Adgre5 exhibited significant differential expression predominantly in capillary endothelial cells (capECs) and vascular endothelial cells (vECs), with markedly reduced expression in the MCAO group compared with the sham group (adjusted *p* < 0.05, logFC < 0) (Figure 2E,F). Given the high consistency between Adgre5 and the results of transcriptomic analysis as well as MR analysis, coupled with its significantly low expression in ischemic stroke as demonstrated by the external validation set (Figure S3), we identified it as the primary research target and focused subsequent analyzes on capECs and vECs.

**TABLE 2:** Differential analysis results of Adgre5 and Msx1 in each cell type.

Symbol	*p*_val	avg_log2FC	pct.1	pct.2	*p*_val_adj	Cell types
Adgre5	0.310	−0.499	0.111	0.147	1	Fibroblast
	0.145	0.189	0.171	0.153	1	Mural cells
	1.91E‐05	−0.572	0.16	0.201	0.376	aEC
	4.15E‐23	−1.244	0.112	0.22	8.17E‐19	vEC
	2.85E‐15	−0.562	0.122	0.166	5.62E‐11	capEC
	0.324	−0.241	0.106	0.116	1	Astrocytes
	0.004	0.282	0.194	0.147	1	Macrophage
	0.271	−0.564	0.153	0.195	1	Neutrophil
	0.116	−0.469	0.204	0.28	1	T cells
Msx1	0.524	0.262	0.309	0.282	1	Fibroblast
	1.74E‐08	−0.280	0.583	0.712	0.0003	Choroid plexus cells
	0.004	−0.381	0.183	0.208	1	aEC

To explore the biological functions associated with Adgre5 in endothelial cells, capECs and vECs from the MCAO group were extracted and stratified into Adgre5‐high and Adgre5‐low groups according to the median expression level of Adgre5. Differential expression analysis was subsequently performed between the two groups, followed by Gene Ontology (GO) enrichment analysis of differentially expressed genes using the clusterProfiler package (v4.16.0) (Figure 2G,H). In capECs, high Adgre5 expression was primarily associated with lipid metabolic regulation, angiogenesis and vascular development, hypoxia response, cell differentiation and migration, cytoskeletal remodeling, and energy metabolism. In vECs, Adgre5 was mainly linked to gene reprogramming, protein localization, energy metabolism, metal ion homeostasis, stress responses, and metabolic adaptation. Collectively, these findings suggest that Adgre5 may contribute to the pathogenesis of ischemic stroke by regulating key endothelial functions involved in vascular homeostasis, metabolic remodeling, and injury responses.

### 3.3 capEC Heterogeneity and Adgre5 Dynamics Along Differentiation

**FIGURE 3:**
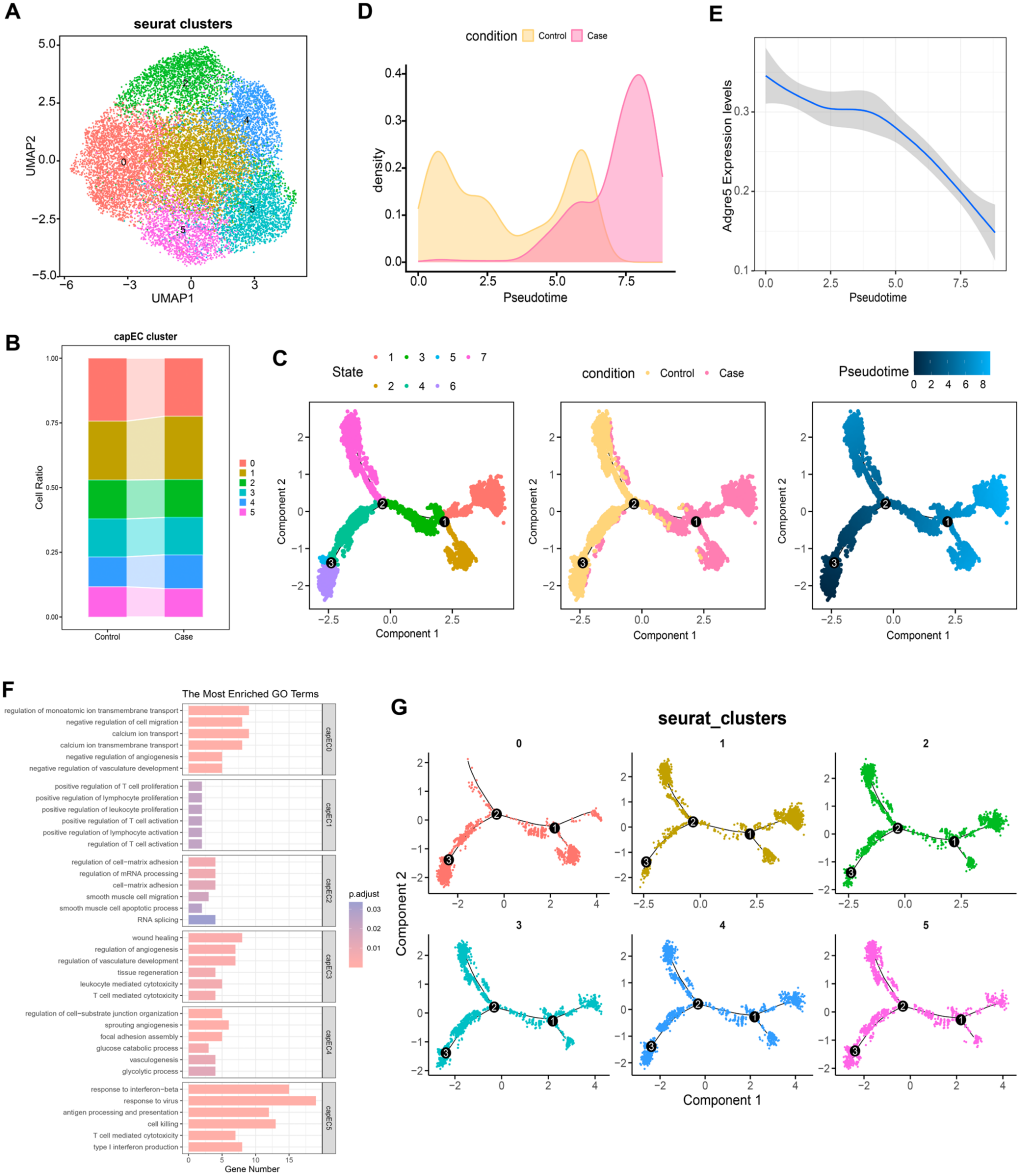
Single‐Cell Trajectory and Functional Annotation of capEC Subtypes. (A) UMAP plot of capEC cell clustering at a resolution of 0.4; (B) Cell proportion distribution of capEC subpopulations between disease and control groups; (C) capEC differentiation trajectory diagram: Left panel shows 7 differentiation states; middle panel depicts distribution of normal and diseased cells along the trajectory; right panel indicates darkest color at differentiation origin and lightest color at differentiation endpoint; (D) Cell density plot; (E) Adgre5 gene expression over simulated time; (F) Enrichment analysis results for each capEC subtype; (G) Distribution of each capEC subtype along the differentiation trajectory.

To elucidate the relationship between capEC heterogeneity and disease progression, we integrated differential abundance, pseudotime trajectory, and functional enrichment analyzes. Six capEC subpopulations were identified (Resolution: 0.5), among which capEC1 and capEC4 were significantly expanded in the disease group, whereas capEC0, capEC2, capEC3, and capEC5 were markedly reduced (Figure 3A,B), indicating profound disease‐associated remodeling of the capillary endothelial compartment.

Pseudotime analysis revealed a continuous transition from normal‐enriched states (States 4–7) to disease‐enriched terminal states (States 1–3) (Figure 3C,D). Notably, Adgre5 was significantly downregulated in multiple capEC populations (capEC0, capEC1, capEC2, capEC3, and capEC5) and progressively decreased along pseudotime (Table 3), reaching its lowest level at the terminal disease‐associated stage (Figure 3E), suggesting a close association between Adgre5 loss and endothelial dysfunction. Subsequently, we further analyzed the differentiation trajectories of its various subpopulations and the functions of their marker genes (Figure 3F,G). Mapping capEC subtypes onto the trajectory demonstrated that capEC0 predominantly occupied the early stage, capEC3, capEC4, and capEC5 were enriched at intermediate stages, whereas capEC1 and capEC2 accumulated at the terminal stage (Figure 3G). Functional annotation further supported this dynamic progression (Figure 3F). Early‐stage capEC0 was enriched in angiogenesis and ion transport pathways, representing a homeostatic endothelial state responsible for maintaining microvascular integrity and blood–brain barrier function. Intermediate‐stage capEC3, capEC4, and capEC5 exhibited enrichment in tissue repair, vascular remodeling, energy metabolism, antigen presentation, and interferon signaling, reflecting an activated endothelial phenotype that responds to ischemic injury and initiates reparative programs. In contrast, terminal‐stage capEC1 and capEC2 were characterized by immune activation, excessive proliferation, cell migration, extracellular matrix remodeling, and apoptotic signaling, indicating a transition from adaptive repair toward maladaptive remodeling and chronic inflammatory dysfunction.

**TABLE 3:** Differential analysis results of Adgre5 in the capEC subtype.

*p*_val	avg_log2FC	pct.1	pct.2	*p*_val_adj	Cell types
1.82E‐05	−0.602	0.133	0.184	0.359	capEC0
0.001	−0.470	0.117	0.152	1	capEC1
0.010	−0.553	0.121	0.157	1	capEC2
3.55E‐05	−0.619	0.129	0.195	0.700	capEC3
0.059	−0.518	0.099	0.124	1	capEC4
0.018	−0.567	0.136	0.173	1	capEC5

Importantly, the disease‐associated expansion of capEC1 and capEC4, together with the depletion of homeostatic capEC0 and other physiological endothelial states, suggests that capECs progressively shift from vascular maintenance toward inflammatory activation, tissue remodeling, and pathological endothelial reprogramming during disease progression. Collectively, these findings indicate that the identified capEC clusters represent distinct functional states along a continuum of endothelial remodeling rather than independent cell lineages, thereby providing a mechanistic explanation for the altered subtype composition observed in diseased tissues.

### 3.4 vEC Heterogeneity and Adgre5 Dynamics Along Differentiation

**FIGURE 4:**
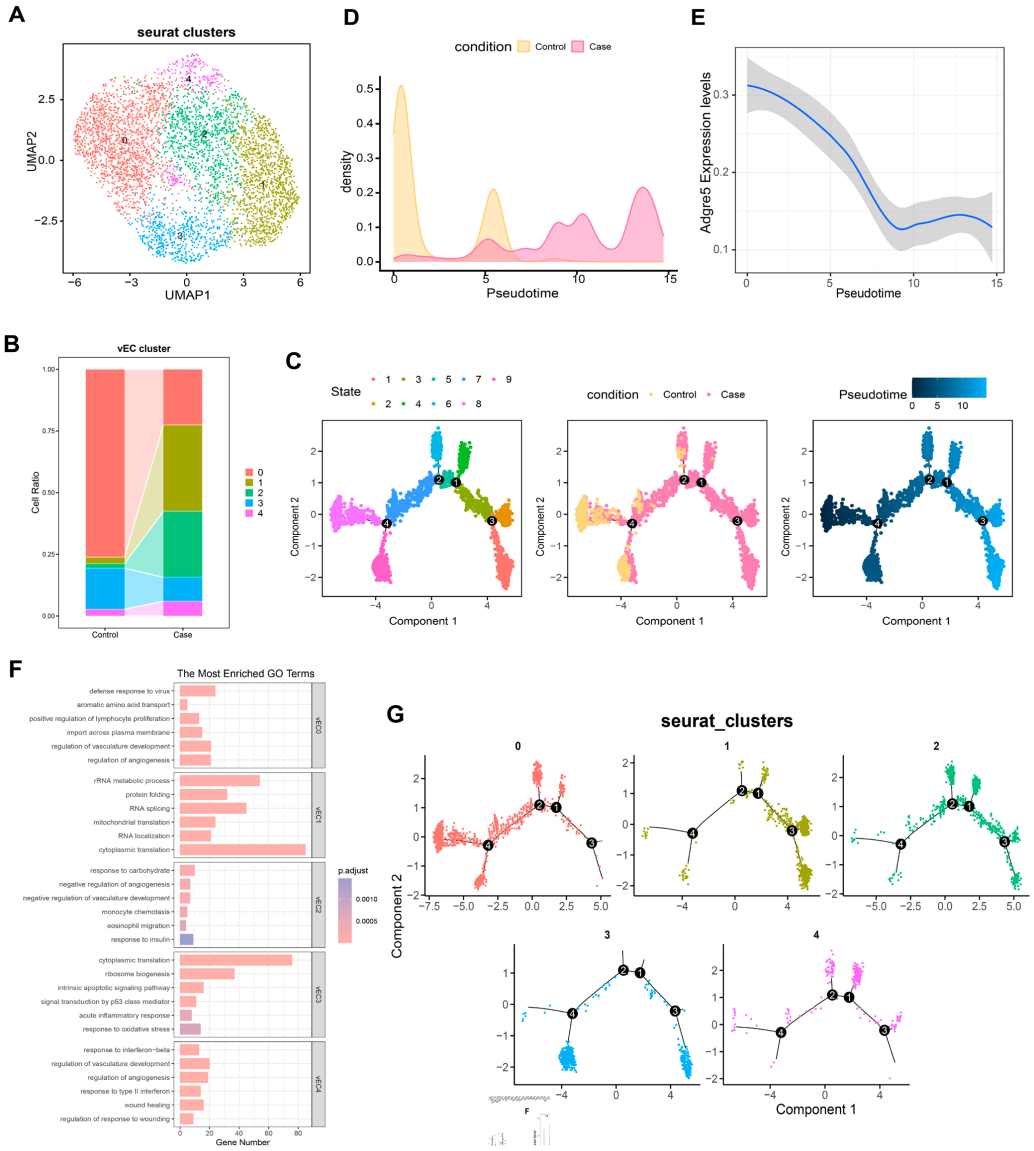
Single‐Cell Trajectory and Functional Annotation of vEC Subtypes. (A) UMAP plot of vEC cell clustering at a resolution of 0.3; (B) Cell proportion distribution of vEC subpopulations between disease and control groups; (C) vEC differentiation trajectory diagram: Left panel shows 9 differentiation states; middle panel depicts distribution of normal and diseased cells along the trajectory; right panel indicates darkest color at differentiation origin and lightest color at differentiation endpoint; (D) Cell density map; (E) Adgre5 gene expression over simulated time; (F) Enrichment analysis results for each vEC subtype; (G) Distribution of each vEC subtype along the differentiation trajectory.

We extracted the vEC cell population and performed integrated dimensionality reduction and clustering analysis on cells from both the control and disease groups. Batch effects across samples were corrected using the RunHarmony function. Using a clustering resolution of 0.3, five distinct cell clusters were identified (Figure 4A). Among these, clusters 0 and 3 exhibited reduced proportions in the disease group, whereas clusters 1, 2, and 4 showed markedly increased proportions compared with the control group (Figure 4B). Differential analysis between normal and diseased states for each vEC subtype revealed (Table 4) that Adgre5 was significantly downregulated in vEC0, vEC1, and vEC3 (logFC < 0).

**TABLE 4:** Differential analysis results of Adgre5 in vEC subtypes.

*p*_val	avg_log2FC	pct.1	pct.2	*p*_val_adj	Cell types
6.42E‐07	−1.017	0.135	0.221	0.013	vEC0
0.0006	−1.752	0.114	0.294	1	vEC1
0.002	−1.383	0.134	0.223	1	vEC3
0.485	−0.694	0.13	0.171	1	vEC4

Pseudo‐temporal analysis identified nine differentiation states of vECs (Figure 4C,D). States 8 and 9 were predominantly composed of control cells and localized at the beginning of the trajectory, whereas states 1–7 were enriched with disease‐derived cells and accumulated toward the terminal branch, indicating a progressive transition from physiological to pathological endothelial states. Consistently, Adgre5 expression gradually declined along pseudotime and reached its lowest level at the disease‐associated terminal stage (Figure 4E), suggesting a potential association between Adgre5 loss and endothelial dysfunction.

To characterize the biological significance of individual vEC states, subtype‐specific marker genes were subjected to GO enrichment analysis (Figure 4F). vEC0, which was predominantly distributed at the early stage of the trajectory, was enriched in angiogenesis, immune surveillance, and substance transport, representing a homeostatic endothelial state responsible for maintaining vascular integrity and normal vascular function. In contrast, vEC1 and vEC3 accumulated during the intermediate stage and were enriched in protein synthesis, inflammatory signaling, stress responses, and apoptotic pathways, indicating endothelial activation in response to injury‐associated inflammatory and hypoxic stimuli. At the late stage of the trajectory, vEC2 and vEC4 became dominant and were characterized by enrichment of chemotaxis, tissue remodeling, interferon responses, wound healing, and vascular remodeling programs, reflecting a transition toward repair‐associated yet progressively dysregulated endothelial states (Figure 4F,G).

Importantly, disease‐associated changes in subtype abundance were highly consistent with the pseudotime trajectory. The reduction of vEC0 and enrichment of vEC1, vEC2, and vEC4 in diseased tissues suggests that endothelial cells progressively exit a homeostatic state and undergo inflammatory activation, tissue repair, and maladaptive remodeling during disease progression. Collectively, these findings indicate that the identified vEC clusters represent distinct functional states along a continuum of endothelial remodeling rather than independent endothelial subtypes, thereby providing a mechanistic link between endothelial heterogeneity and disease progression.

### 3.5 LAMININ and JAM Signaling Mediate Adgre5‐Dependent Intercellular Communication in vEC

**FIGURE 5:**
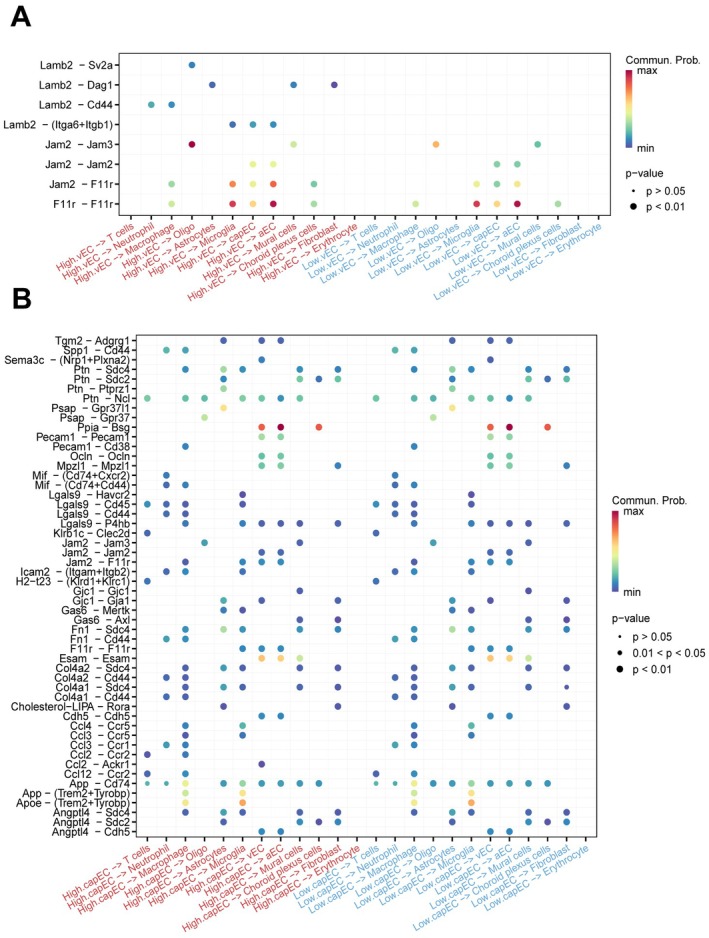
Comparative Analysis of Cell–Cell Communication Mediated by Adgre5‐High and ‐Low vEC and capEC. (A) Communication between vEC with high and low Adgre5 expression and other cell types; (B) Communication between capEC with high and low Adgre5 expression and other cell types.

Examining communication between Adgre5‐high vECs and other cell types (Figure 5A), we observed that the LAMININ signaling pathway emerged exclusively during interactions between Adgre5‐high vECs and other cell types. For instance: interacting with neutrophils and macrophages via the Lamb2‐Cd44 receptor pair; and interacting with astrocytes, parietal cells, and fibroblasts via the Lamb2‐Dag1 receptor pair. Furthermore, The JAM signaling pathway also exhibits stronger interactions between Adgre5‐high vECs and other cell types. However, examining communication interactions between Adgre5‐high and Adgre5‐low capECs and other cell types reveals no significant differences in communication between the two groups (Figure 5B). Given that the excessive recruitment and activation of peripheral immune cells are recognized as key drivers of secondary neuroinflammation and tissue damage (Duan et al. 2024; Shichita et al. 2023), we infer that the Lamb2‐CD44 signaling axis is more directly associated with the inflammatory pathological processes underlying disease progression. Furthermore, CD44 has been widely recognized as a critical regulatory factor governing leukocyte adhesion, migration, and inflammatory activation (Johnson et al. 2000; Johnson and Ruffell 2009; McDonald and Kubes 2015). Compared to other predicted receptor combinations, it provides a more robust mechanistic basis for experimental validation. Therefore, we will prioritize the Lamb2‐CD44 signaling pathway as a representative and biologically relevant model pathway for downstream functional studies.

In summary, Adgre5 is a key vEC regulatory molecule in the IS. In disease states, reduced Adgre5 expression may weaken vEC communication networks with immune cells and glial cells, thereby impairing tissue repair, inflammatory regulation, and restoration of neural function.

### 3.6 JUNB Is a Key Upstream Regulator of Adgre5 in vEC

**FIGURE 6:**
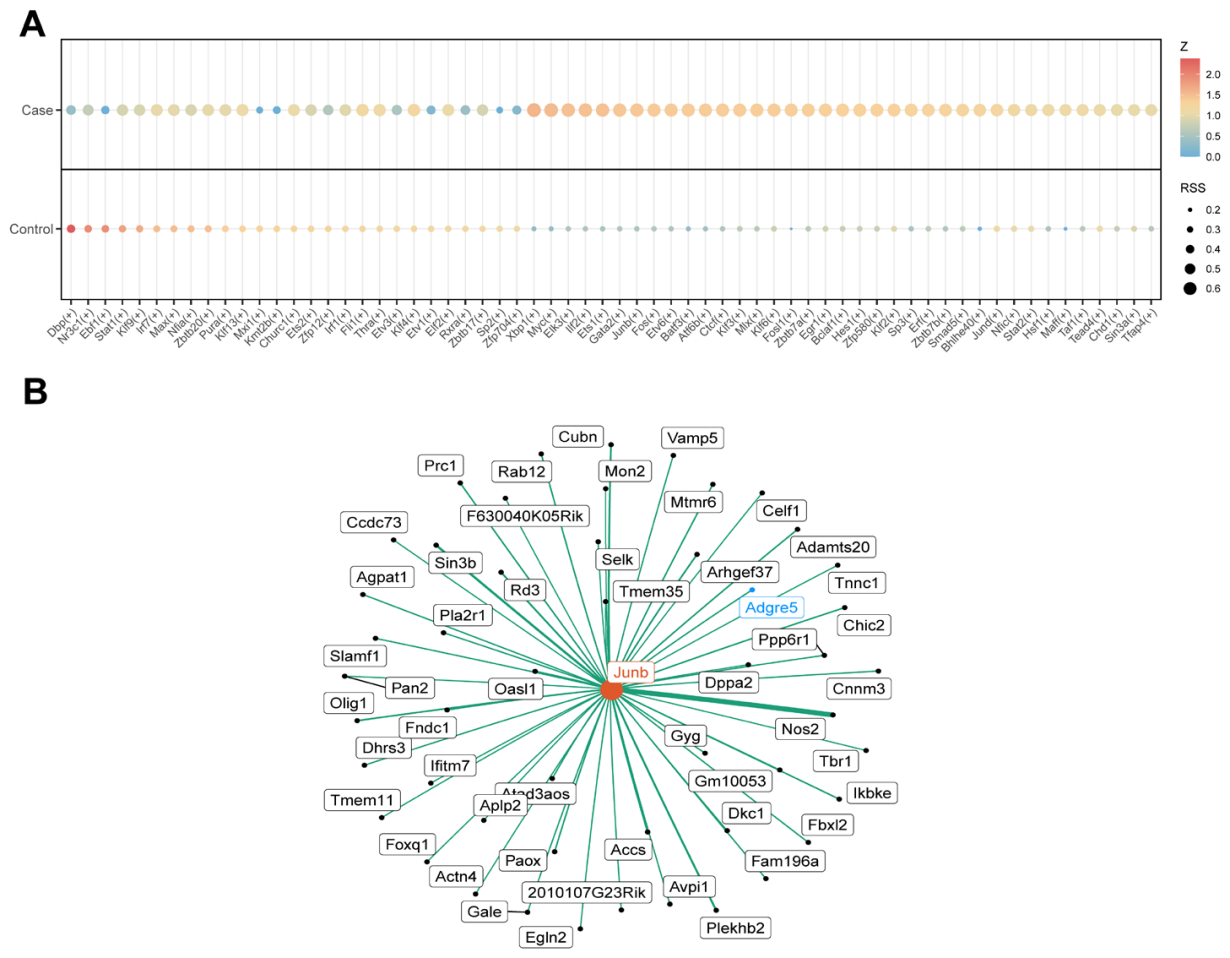
Identification of Disease‐Specific Transcription Factors and the Regulatory Network of JUNB. (A) Normal versus disease‐specific regulatory factors; (B) Regulatory network diagram of the Junb transcription factor.

Based on MR and transcriptome differential analysis results, we identified Adgre5 as a protective factor against ischemic stroke. Therefore, further investigation into Adgre5's key upstream regulatory factors that can be targeted pharmacologically to prevent disease onset holds significant biological importance. To identify transcription factors regulating Adgre5, we extracted vEC data for pySCENIC transcription factor analysis, identifying 37 disease‐specific transcription factors (Figure 6A). We screened for transcription factors significantly upregulated in diseased vECs using the criteria *p*.adj < 0.05 and logFC > 0.1, identifying Junb, Fos, Ilf2, Egr1, Bhlhe40, Xbp1, Maff, and Myc (Table 5).

**TABLE 5:** Differential analysis results of disease‐specific regulatory transcription factors in vEC between case and control groups.

Symbol	*p*_val	avg_log2FC	pct.1	pct.2	*p*_val_adj
Junb	3.69E‐17	0.341	0.915	0.813	7.27E‐13
Fos	3.16E‐29	0.483	0.858	0.72	6.22E‐25
Ilf2	2.59E‐10	0.552	0.305	0.21	5.10E‐06
Egr1	9.77E‐40	0.701	0.575	0.343	1.92E‐35
Bhlhe40	2.18E‐17	0.767	0.331	0.201	4.29E‐13
Xbp1	2.25E‐84	1.167	0.774	0.547	4.44E‐80
Maff	3.12E‐23	1.248	0.257	0.123	6.15E‐19
Myc	6.28E‐27	3.016	0.112	0.013	1.24E‐22

Subsequently, we extracted vEC disease data and divided samples into high‐ and low‐expression groups based on median Adgre5 expression for differential analysis. Genes significantly upregulated in the low‐expression group were selected as potential key transcription factors regulating Adgre5 (Table 6). Combining this with gene–gene co‐expression networks inferred by the GRNboost2 algorithm between transcription factors and their potential targets, we identified transcription factors regulating Adgre5.

**TABLE 6:** Results of high vs. low differential analysis in the vEC disease group.

Symbol	*p*_val	avg_log2FC	pct.1	pct.2	*p*_val_adj
Fos	0.354	−0.108	0.855	0.858	1
Junb	0.010	−0.112	0.907	0.916	1
Maff	0.368	−0.295	0.253	0.258	1
Xbp1	0.0004	−0.333	0.752	0.777	1

Furthermore, the ChEA analysis integrating multiple databases (ARCHS4, ReMap, GTEx) still confirms a regulatory relationship between JUNB and ADGRE5 (Table S2). Ultimately, Junb was found to exert significant regulatory influence on Adgre5 (Figure 6B).

### 3.7 Molecular Docking Analysis of SN‐011 and JUNB

**FIGURE 7:**
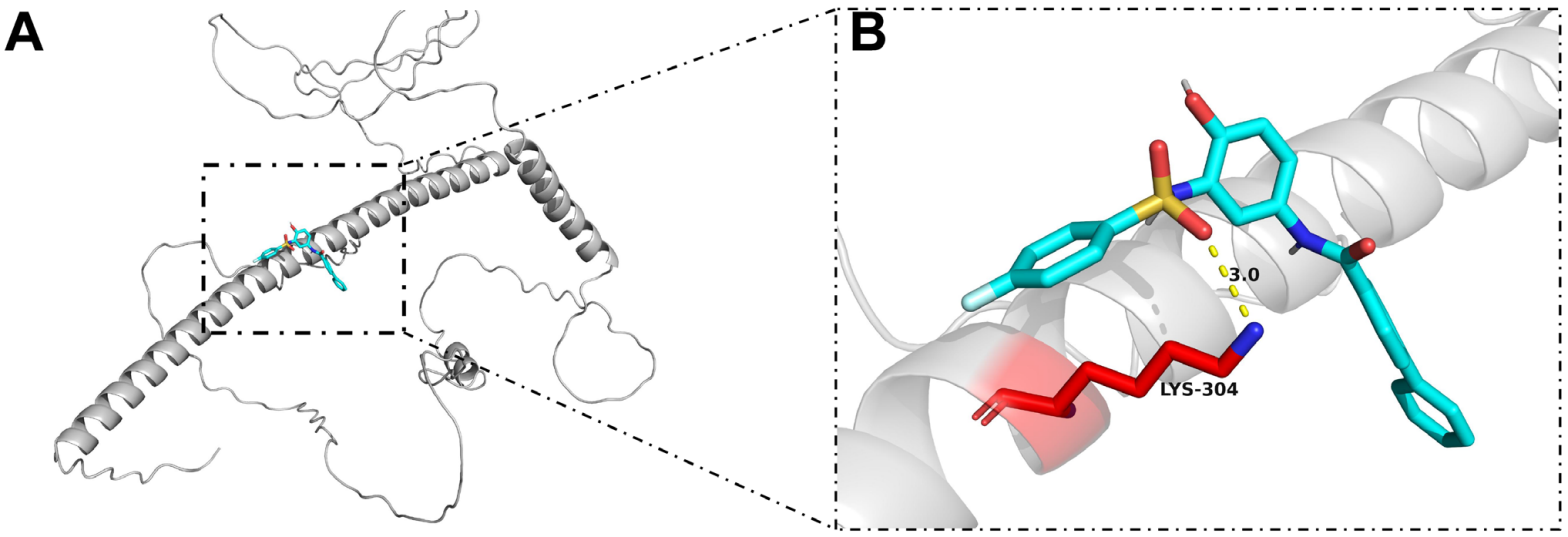
SN‐011 Docking with JUNB Protein. (A) Specific docking position information of SN‐011 within the JUNB protein structure; (B) Molecular docking results of SN‐011 with JUNB protein.

To investigate whether the STING inhibitor SN‐011 can bind to the JUNB protein and exert an effect, we retrieved the structural data of SN‐011 from the PubChem database and performed molecular docking using Autodock Vina. A lower binding energy indicates a more stable interaction between the ligand and receptor. The molecular docking results showed that the binding energy between SN‐011 and the JUNB protein was −6.8 kcal/mol (Table 7), which is less than −5 kcal/mol, suggesting a strong binding affinity. SN‐011 may form a hydrogen bond with Lys304, a key amino acid residue of the JUNB protein (Figure 7A,B).

**TABLE 7:** Molecular docking results.

Protein	Drug	Top5 energy (kcal/mol)
JUNB	SN‐011	−6.8
		−6.8
		−6.7
		−6.6
		−6.4

### 3.8 SN‐011 Alleviates Post‐Ischemic Inflammatory Response by Inhibiting JUNB to Upregulate ADGRE5 and Activate the Lamb2–Cd44 Ligand–Receptor Axis

**FIGURE 8:**
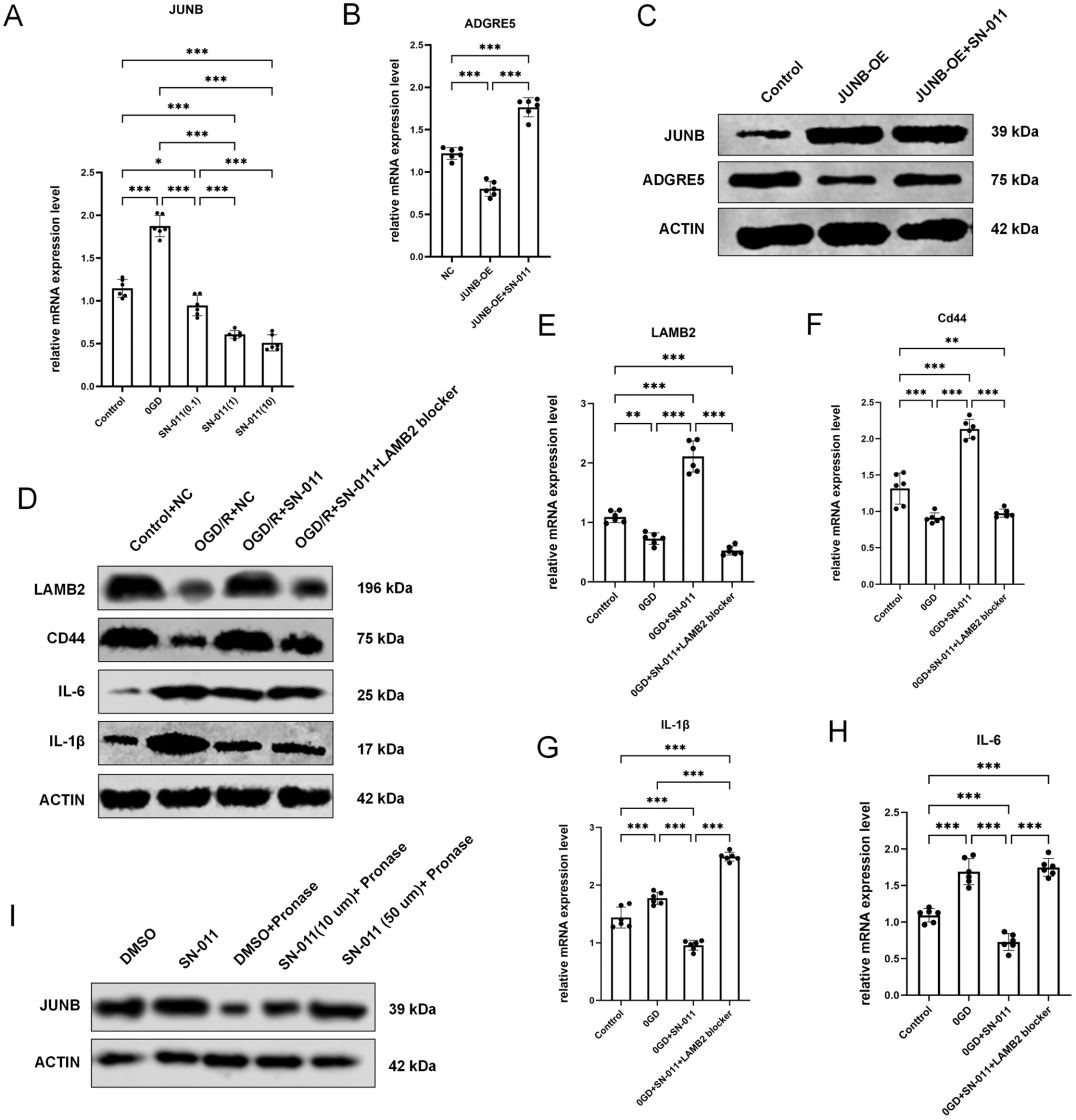
SN‐011 suppresses JUNB and restores the ADGRE5–LAMB2/CD44 signaling pathway in OGD‐injured endothelial cells. (A) Regulatory effect of the STING inhibitor SN‐011 on the transcription factor JUNB; (B) Transcriptional regulation of ADGRE5 by JUNB; (C) Western blot analysis detect the protein expression levels of ADGRE5 following JUNB overexpression and SN‐011 intervention; (D) Western blot analysis of Lamb2, Cd44, IL‐1β, and IL‐6 protein levels in the Control, OGD, OGD+SN‐011, and OGD+SN‐011+LAMB2 blocker groups. (E–H) qPCR analysis of Lamb2 (E), Cd44 (F), IL‐1β (G), and IL‐6 (H) expression in the indicated groups; (I) DARTS‐Western blot assay showing the effect of SN‐011 on JUNB protease resistance. Cell lysates were incubated with DMSO or increasing concentrations of SN‐011 (10 and 50 μM) prior to Pronase digestion. JUNB protein abundance was subsequently determined by Western blotting. Statistical significance was defined as follows: Ns, not significant; *p* < 0.05; *p < 0.05; **p < 0.01; ***p < 0.001; ****p < 0.0001.

To investigate the regulatory effect of the STING inhibitor SN‐011 on the transcription factor JUNB, primary human brain microvascular endothelial cells (HBMECs) were first subjected to oxygen–glucose deprivation (OGD) to model ischemic injury. qPCR results showed that OGD treatment significantly upregulated the mRNA expression level of JUNB compared with the control group (*p* < 0.01). Following treatment with different concentrations of SN‐011 (0.1, 1, 10 μM) for 24 h, JUNB expression decreased in a concentration‐dependent manner, indicating that SN‐011 effectively inhibits the OGD‐induced aberrant upregulation of JUNB (Figure 8A).

To verify the transcriptional regulatory relationship of JUNB on ADGRE5, HBMECs were used to establish JUNB overexpression (JUNB‐OE) and JUNB‐OE combined with SN‐011 treatment models. qPCR and Western blot results revealed that compared with the negative control (NC) group, the mRNA and protein expression level of ADGRE5 was significantly decreased in the JUNB‐OE group (*p* < 0.01); however, upon SN‐011 treatment in the JUNB‐OE background, ADGRE5 expression was markedly restored (Figure 8B,C). These results indicate that JUNB negatively regulates ADGRE5 transcription, and SN‐011 can relieve this inhibitory effect and restore ADGRE5 expression.

To further clarify the effect of SN‐011 on the interaction between vascular endothelial cells and neutrophils, a co‐culture system of HBMECs and neutrophils was established, and the expression of the Lamb2–Cd44 ligand–receptor pair and related inflammatory factors was examined. The results showed that compared with the control group, OGD treatment significantly downregulated the mRNA and protein expression levels of LAMB2 and Cd44 (*p* < 0.05), while the expression levels of the inflammatory factors IL‐6 and IL‐1β were significantly increased (*p* < 0.01). Following OGD combined with SN‐011 treatment, the expression of LAMB2 and Cd44 was significantly restored (*p* < 0.01), accompanied by a marked decrease in IL‐6 and IL‐1β expression levels (*p* < 0.01). Upon administration of a LAMB2 blocker in the OGD+SN‐011 group, Cd44 expression decreased again, while IL‐6 and IL‐1β expression levels increased significantly (*p* < 0.01) (Figure 8D–H).

To investigate whether SN‐011 directly interacts with JUNB, a DARTS assay coupled with Western blotting was performed (Figure 8D–I). Pronase treatment markedly reduced JUNB protein levels in the DMSO control group, indicating efficient proteolytic degradation. In contrast, SN‐011 treatment significantly attenuated Pronase‐induced JUNB degradation, as evidenced by the increased intensity of JUNB bands under Pronase digestion. This protective effect appeared to be dose‐dependent, with stronger preservation observed at 50 μM than at 10 μM SN‐011. In comparison, ACTIN levels remained largely unchanged among groups. These findings suggest that SN‐011 enhances the protease resistance of JUNB, supporting a potential direct interaction between SN‐011 and JUNB.

Collectively, these findings suggest that SN‐011 may suppress the inflammatory interaction between endothelial cells and neutrophils by upregulating the Lamb2–Cd44 ligand–receptor signal mediated by the JUNB‐ADGRE5 axis, thereby alleviating post‐ischemic inflammatory injury.

## 4 Discussion

This study integrates transcriptomic differential analysis, Mendelian randomization, single‐cell transcriptomics, and molecular docking techniques to reveal for the first time that ADGRE5 serves as a key protective gene in ischemic stroke. Its downregulation in venous endothelial cells may impair vascular repair and immune regulation by weakening intercellular communication mediated through the LAMININ and JAM signaling pathways. More importantly, we discovered that the STING inhibitor SN‐011 may restore neuroprotection by targeting the transcription factor JUNB to release its transcriptional repression on ADGRE5, thereby reconstructing the vEC‐centered cellular communication network.

Mendelian randomization combined with differential analysis revealed a significant negative correlation between ADGRE5 expression levels and ischemic stroke risk (OR < 1), suggesting its potential as a protective factor against IS. Adhesion G protein‐coupled receptor 5 (ADGRE5), also known as differentiation cluster 97 (CD97), belongs to the seven‐transmembrane epidermal growth factor (EGF) subfamily of class B G protein‐coupled receptors (GPCRs) (Wang et al. 2005; Ward et al. 2013). This molecule was initially identified as an early activation marker in lymphocytes, highly expressed on granulocytes and monocytes/macrophages, and upregulated during inflammatory responses (Liu et al. 2024; Eichler et al. 1997; Martin et al. 2019; Wang et al. 2016). It promotes angiogenesis by interacting with ligands such as CD55 and integrins, as well as through adhesion interactions with extracellular matrix components (Moreno‐Salinas et al. 2025; Langenhan et al. 2013; Stacey et al. 2000). Directed in vivo angiogenesis assay (DIVAA) using purified proteins revealed that CD97α promotes angiogenesis in vivo (Wang et al. 2005). Furthermore, a study in diabetic patients' endothelial cells demonstrated that overexpression of CD97 ameliorates high glucose‐induced endothelial cell migration dysfunction (Zhao et al. 2017). Collectively, these data highlight the pro‐angiogenic function of ADGRE5 in endothelial cells.

Single‐cell analysis revealed that ADGRE5 was significantly downregulated in vascular endothelial cells, with its expression levels progressively decreasing during cellular differentiation. Pseudo‐time analysis further demonstrated a sustained decline in ADGRE5 expression as vECs transitioned from early differentiation (functional equilibrium state) to late differentiation (inflammatory dysregulation and apoptotic state). This dynamic pattern suggests that ADGRE5 downregulation may represent a key molecular event in vEC functional inactivation, closely linked to impaired vascular repair following ischemia.

Cell communication analysis revealed that vECs overexpressing ADGRE5 establish extensive interaction networks with multiple cell types via the LAMININ signaling pathway. Involved receptor pairs include Lamb2‐CD44, Lamb2‐Itga6+Itgb1, and Lamb2‐Dag1. Notably, Lamb2‐CD44 primarily mediates communication between endothelial cells and infiltrating immune cells such as neutrophils and macrophages, whereas Lamb2‐Dag1 is more involved in interactions between endothelial cells and astrocytes, pericytes, and fibroblasts. These findings suggest that ADGRE5 may not only contribute to maintaining vascular wall structural homeostasis but also influence post‐ischemic brain inflammation and repair processes by regulating signal exchange among distinct cell populations within neurovascular units. As a core component of the basement membrane, LAMININ provides structural support for endothelial cells and regulates cell adhesion, migration, and survival by binding to cell surface receptors such as integrins (e.g., the β1 integrin family) and CD44 (Zhao et al. 2025; Vivinus‐Nebot et al. 2004; Timpl 1996; Nonnast et al. 2025; Simon‐Assmann et al. 2011; Marchand et al. 2019). The JAM signaling pathway participates in maintaining tight junctions between cells and the blood–brain barrier (Ebnet 2017; Jia et al. 2013; Hallada et al. 2024; Schottlaender et al. 2020). In summary, ADGRE5 downregulation may disrupt coordinated interactions between vECs and surrounding cells by weakening key signaling pathways mediated by LAMININ and JAM, leading to impaired vascular repair and persistent neuroinflammation.

Notably, the Lamb2‐CD44 signaling axis primarily mediates communication between vascular endothelial cells and infiltrating immune cells. Excessive recruitment and persistent activation of peripheral immune cells are widely recognized as major drivers of secondary neuroinflammation and tissue damage following ischemic stroke. Moreover, CD44 has been extensively implicated in leukocyte adhesion, transendothelial migration, and inflammatory activation (Johnson et al. 2000; Johnson and Ruffell 2009; McDonald and Kubes 2015). Therefore, compared with the other predicted receptor combinations, the Lamb2‐CD44 axis is more likely to be directly involved in the establishment and maintenance of the post‐stroke neuroinflammatory microenvironment.

Based on this rationale, we further validated the Lamb2‐CD44 signaling axis in an oxygen–glucose deprivation (OGD)‐induced human brain microvascular endothelial cell (HBMEC) injury model. We found that the expression of both Lamb2 and CD44 was significantly reduced under ischemic–hypoxic conditions, accompanied by a marked increase in the pro‐inflammatory cytokines IL‐1β and IL‐6. These findings suggest that ischemic injury may impair Lamb2‐CD44‐mediated endothelial communication, thereby compromising neurovascular unit homeostasis and tissue repair capacity, ultimately facilitating the amplification and persistence of inflammatory responses. In conjunction with our single‐cell transcriptomic analyzes, we further speculate that the downregulation of ADGRE5 may represent an upstream event in this pathological process. By disrupting Lamb2‐CD44 signaling, reduced ADGRE5 expression may contribute to endothelial dysfunction, aberrant immune cell recruitment, and exacerbated neuroinflammation, thereby promoting the progression of ischemic stroke.

To investigate the upstream regulatory mechanisms of ADGRE5, we identified the transcription factor JUNB—potentially a target regulator of ADGRE5—through pySCENIC analysis combined with GRNboost2 co‐expression networks. As a transcription factor, the JUNB proto‐oncogene is a component of the AP‐1 dimer complex and participates in regulating cell differentiation, apoptosis, and inflammatory responses (Fontana et al. 2015). Previous studies demonstrated that in ischemic stroke models, the JUNB/NF‐κB pathway regulates M1/M2 polarization of microglia, influencing neuroinflammatory processes (Xie et al. 2025). Another study found that following middle cerebral artery occlusion, JUNB mRNA expression was induced in the cerebral cortex, thalamus, basal ganglia, and hippocampus (Kinouchi et al. 1994). Furthermore, we validated the binding affinity between JUNB and SN‐011 through molecular docking and DARTS‐WB assay, suggesting SN‐011 may directly target JUNB, thereby influencing its transcriptional regulation of ADGRE5. Integrating these findings, we propose the SN‐011‐JUNB‐ADGRE5 regulatory axis hypothesis: During the acute phase of ischemic stroke, JUNB is rapidly induced, negatively regulating ADGRE5 expression. This leads to downregulation of ADGRE5 in vECs, impairing intercellular communication mediated by the LAMININ signaling pathway. This model is supported by our in vitro observations showing that SN‐011 not only directly inhibited JUNB expression in OGD‐injured endothelial cells, but also restored ADGRE5 expression suppressed by JUNB overexpression. Furthermore, SN‐011 effectively rescued the OGD‐induced downregulation of LAMB2 and CD44 while simultaneously reducing the expression of the pro‐inflammatory cytokines IL‐6 and IL‐1β. Notably, blockade of LAMB2 abolished these protective effects, resulting in reduced CD44 expression accompanied by a marked increase in IL‐6 and IL‐1β levels. These findings suggest that the anti‐inflammatory and endothelial‐protective effects of SN‐011 are at least partially mediated through restoration of the LAMB2‐CD44 signaling pathway.

Taken together, our data indicate that ischemia‐induced JUNB activation may contribute to endothelial dysfunction through repression of ADGRE5 and subsequent disruption of laminin‐dependent cell–cell communication. Pharmacological intervention with SN‐011 appears to counteract this process, thereby preserving neurovascular unit homeostasis and attenuating inflammatory injury. Although the precise molecular interactions require further investigation, the SN‐011–JUNB–ADGRE5–LAMB2/CD44 axis may represent a previously unrecognized mechanism underlying neurovascular remodeling after ischemic stroke.

This study holds significant implications. First, it identifies ADGRE5 as a protective gene in IS for the first time, providing a novel target for studying the genetic mechanisms of IS. Second, it reveals the dynamic expression of ADGRE5 in vECs at the single‐cell level and its regulation of the cell communication network, deepening our understanding of impaired vascular repair after ischemia. Finally, it proposes the SN‐011‐JUNB‐ADGRE5 regulatory axis, providing a novel molecular mechanism for SN‐011 treatment of IS and opening new avenues for therapeutic strategies targeting vascular endothelial cells.

Our study still has certain limitations. (1) Our mechanistic conclusions are limited to the experimentally validated Lamb2‐CD44 signaling axis, and the involvement of other predicted pathways requires further confirmation. (2) The findings presented here represent exploratory conclusions based solely on the available dataset and warrant validation through larger‐scale cohort studies and experimental investigations. (3) The binding site between the transcription factor JUNB and ADGRE5 has not yet been fully elucidated in this study and will require additional experimental verification in future research.

## 5 Conclusion

In summary, SN‐011 targets the JUNB protein, inhibiting its negative regulation of the downstream gene ADGRE5. This leads to the upregulation of ADGRE5 expression and the activation of its function, driving vEC to establish a broad and coordinated cellular communication network centered on the LAMININ and JAM pathways. Consequently, this network regulates cell adhesion, migration, proliferation, and apoptosis, promoting vascular repair, immune regulation, neuroprotection, and tissue remodeling. These findings provide a theoretical basis for the development of the STING inhibitor SN‐011 as a therapeutic agent for ischemic stroke.

## Author Contributions

**ZunKe Gong:** conceptualization, investigation, funding acquisition, methodology, software, project administration, resources, supervision. **XiangLing Ou:** validation, visualization, writing – review and editing, writing – original draft, data curation, formal analysis.

## Funding

The authors have nothing to report.

## Disclosure

Generative AI Statement: The authors confirm that no generative AI tools were used in the preparation of this manuscript.

## Ethics Statement

This study utilized commercially available primary human brain microvascular endothelial cells (HBMECs) and commercially sourced mouse neutrophils. No human subjects or animal experiments were involved; therefore, ethical approval was not required.

## Conflicts of Interest

The authors declare no conflicts of interest.

## Supporting information


**Figure S1:** Visualization of PCA principal component analysis results from transcriptome sequencing.
**Figure S2:** Single‐cell sequencing analysis to eliminate batch effects. (A) PCA dimensionality reduction plot before batch processing. (B) Harmony's dimensionality‐reduced graph after batch processing.
**Figure S3:** The independent GEO dataset GSE183439 (containing 4 sham‐operated samples and 4 stroke model samples) was used for external validation of the expression trends of ADGRE5.

**Table S1:** The Propeller method was used to analyze differences in the composition of cell types.

**Table S2:** The ChEA analysis integrating multiple databases (ARCHS4, ReMap, GTEx) identified the transcription factors that regulate ADGRE5 expression.

## Data Availability

The data that support the findings of this study are available on request from the corresponding author. The data are not publicly available due to privacy or ethical restrictions.
