# Supplementary material for: Multi‐Omics Integration Reveals That SN‐011 Targets JUNB to Upregulate ADGRE5 and Restore Vascular Endothelial Cell Communication in Ischemic Stroke
Source: Chem Biol Drug Des. 2026 Aug 4;108(2):e70363. doi: 10.1111/cbdd.70363 (PMC13435820; doi:10.1111/cbdd.70363)
Supplement: Supplementary file 1 [file CBDD-108-e70363-s003.docx]

**Supplementary Figure 1. Visualization of PCA principal component analysis results from transcriptome sequencing.**

**Supplementary Figure 2. Single-cell sequencing analysis to eliminate batch effects. (A)** PCA Dimensionality Reduction Plot Before Batch Processing. **(B)** Harmony's dimensionality-reduced graph after batch processing.

**Supplementary Figure 3. The independent GEO dataset GSE183439 (containing 4 sham-operated samples and 4 stroke model samples) was used for external validation of the expression trends of ADGRE5.**

**Supplementary Table 1. The Propeller method was used to analyze differences in the composition of cell types.**

Note: PropMean.

Case: Average proportion of this cell type in the disease group;

PropMean-Control: Average proportion of this cell type in the normal group;

Propratio: Ratio (Case/Control);>1 indicates enrichment in the case group.

**Supplementary Table 2. The ChEA analysis integrating multiple databases (ARCHS4, ReMap, GTEx) identified the transcription factors that regulate ADGRE5 expression.**
